# AI-enhanced adaptive testing with cognitive diagnostic feedback and its association with performance in undergraduate surgical education: a pilot study

**DOI:** 10.3389/fnbeh.2025.1735237

**Published:** 2026-01-06

**Authors:** Nuno Silva Gonçalves, Carlos Collares, José Miguel Pêgo

**Affiliations:** 1 Life and Health Sciences Research Institute (ICVS), University of Minho, Braga, Portugal; 2 ICVS/3B’s, PT Government Associate Laboratory, Braga, Portugal; 3 European Board of Medical Assessors, Cardiff, United Kingdom; 4 Inspirali Educação, São Paulo, Brazil; 5 Faculdades Pequeno Príncipe, Curitiba, Brazil; 6 Medical Education Unit, Faculty of Medicine and Biomedical Sciences, University of Algarve, Faro, Portugal; 7 iCognitus4ALL – IT Solutions, Porto, Portugal

**Keywords:** artificial intelligence, computerized adaptive testing, cognitive diagnostic modeling, surgical education, feedback, cognitive skills, assessment innovation, educational technology

## Abstract

**Background:**

Effective feedback in the cognitive domain is essential for surgical education but often limited by resource constraints and traditional assessment formats. Artificial Intelligence (AI) has emerged as a catalyst for innovation, enabling automated feedback, real-time cognitive diagnostics, and scalable item generation, thereby transforming how future surgeons learn and are assessed.

**Methods:**

An item bank of 150 multiple-choice questions was developed using AI-assisted item generation and difficulty estimation. A formative Computerized Adaptive Testing (CAT), balanced across three cognitive domains (memory, analysis, and decision) and surgical topics, was delivered via QuizOne^®^ 3–5 days before the summative Progress Test. A total of 147 students participated, of whom 116 completed the formative CAT. Performance correlations, group comparisons, analysis of covariance (ANCOVA), and regression analyses were conducted.

**Results:**

Students who voluntarily completed CAT showed higher Progress Test scores, though causality cannot be established due to self-selection bias (*p* = 0.021), with the effect persisting after adjusting for prior academic performance (ANCOVA *p* = 0.041). Memory skills were the strongest predictors of summative outcomes (*R*^2^ = 0.180, *β* = 0.425), followed by analysis (*R*^2^ = 0.080, *β* = 0.283); decision was not significant (*R*^2^ = 0.029, *β* = 0.170).

**Conclusion:**

AI-enhanced CAT–Cognitive Diagnostic Modeling (CDM) represents a promising formative approach in undergraduate surgical education, being associated with higher summative performance and providing individualized diagnostic feedback. Refining feedback presentation and enhancing decision-making assessment could further optimize its educational impact.

## Introduction

Artificial Intelligence (AI) is transforming medical education by enabling automated item generation, difficulty estimation, and individualized feedback that was previously impractical (Pohn et al., 2025; Shaw et al., 2025; Gordon et al., 2024; Mir et al., 2023). When integrated with adaptive testing and cognitive diagnostic models, AI allows feedback to evolve from static score reporting into a continuous, formative process that guides learning (Sunmboye et al., 2025). This approach is particularly valuable in surgical education, where cognitive, technical, and non-technical skills intersect and are required, allowing educators to identify specific strengths and weaknesses and promote deliberate practice toward surgical competence (Gomez et al., 2025; Rosendal et al., 2023; Ounounou et al., 2019; Dedy et al., 2013).

Although feedback is widely recognized as a key driver of learning, its application in the cognitive domain, particularly during undergraduate surgical education, remains underexplored (Garner et al., 2014; El Boghdady and Alijani, 2017). Traditional mechanisms often fail to provide timely, specific, and actionable insights into students’ cognitive performance, especially in complex domains such as reasoning and decision-making (Burgess et al., 2020; Shaughness et al., 2017). As medical curricula increasingly adopt competency-based models, adopting assessment strategies that deliver targeted, data-informed feedback has become essential to enhance learning and self-regulation (Ross et al., 2022).

Cognitive competence involves integrating basic scientific knowledge with clinical information to interpret findings and make informed decisions under uncertainty, skills that are critical in surgical practice (Crebbin et al., 2013; Madani et al., 2017). However, traditional multiple-choice exams often emphasize factual recall, provide delayed feedback, and fail to capture deeper levels of reasoning (Butler and Roediger, 2008).

Providing feedback in summative assessments remains a challenge in medical education. Concerns over item security, fairness, and resource limitations often lead institutions to restrict item-level feedback (Appelhaus et al., 2023; Harrison, 2017). Consequently, summative assessments frequently become “black boxes,” offering scores without meaningful guidance and reinforcing a culture of performance rather than development.

When integrated into assessment systems, AI can support the creation of high-quality questions, estimate item difficulty, and generate individualized diagnostic feedback with minimal faculty effort. These capabilities complement Computerized Adaptive Testing (CAT), a psychometric method proposed by Lord (1971), Owen (1975), and Chang and Ying (1996) that dynamically adjusts item difficulty based on student responses (Chang and Ying, 1996). By tailoring the test to the learner’s ability level, CAT increases the efficiency and precision of assessment with fewer items, reducing test fatigue while maximizing information. It can also be useful in determining the true score and competence of an examinee (Collares and Cecilio-Fernandes, 2019; Burr et al., 2016). In the context of medical education, CAT has been successfully implemented in progress testing, licensing exams, and residency selection processes (Burr et al., 2016; Xu et al., 2023; Van Wijk et al., n.d.; Seo et al., 2024). Its potential as a formative tool for learning, however, remains underutilized.

CAT can also be coupled with Cognitive Diagnostic Modeling (CDM), a psychometric framework that analyzes student responses to infer the proficiency of specific cognitive attributes. Unlike classical test theory, which provides a single overall score, CDM enables a multidimensional understanding of performance by categorizing items and responses according to specific cognitive processes. For instance, an item might assess recall of factual knowledge, interpretation of clinical signs, or the application of pathophysiological reasoning. By classifying and analyzing items in this way, CDM supports the generation of individualized feedback, offering students a roadmap for targeted improvement (Ma et al., 2023; Williamson, n.d.; Leighton and Gierl, 2007; Barthakur et al., 2022). This granular diagnostic capability is particularly relevant in complex curricular areas like surgery, where different cognitive skills are needed to approach diverse clinical scenarios. In this study, items were categorized into three cognitive diagnostic models (memory, analysis, and decision) reflecting the cognitive tasks proposed by the National Board of Medical Examiners (NBME) for assessing medical knowledge application (Billings et al., n.d.). These domains were selected because they align with the NBME’s framework for evaluating progressively complex levels of cognitive processing, ranging from factual recall to clinical reasoning and decision-making, which are particularly relevant in the context of surgical education.

Despite their theoretical advantages, CAT and CDM have rarely been explored as learning tools in undergraduate surgical education. By evaluating an AI-supported CAT–CDM intervention in a real educational setting, this study aims to advance data-informed feedback practices, improve alignment between formative and summative assessment, and support the development of self-regulated surgical learners.

## Materials and methods

### Item development and cognitive models

**Figure 1:**
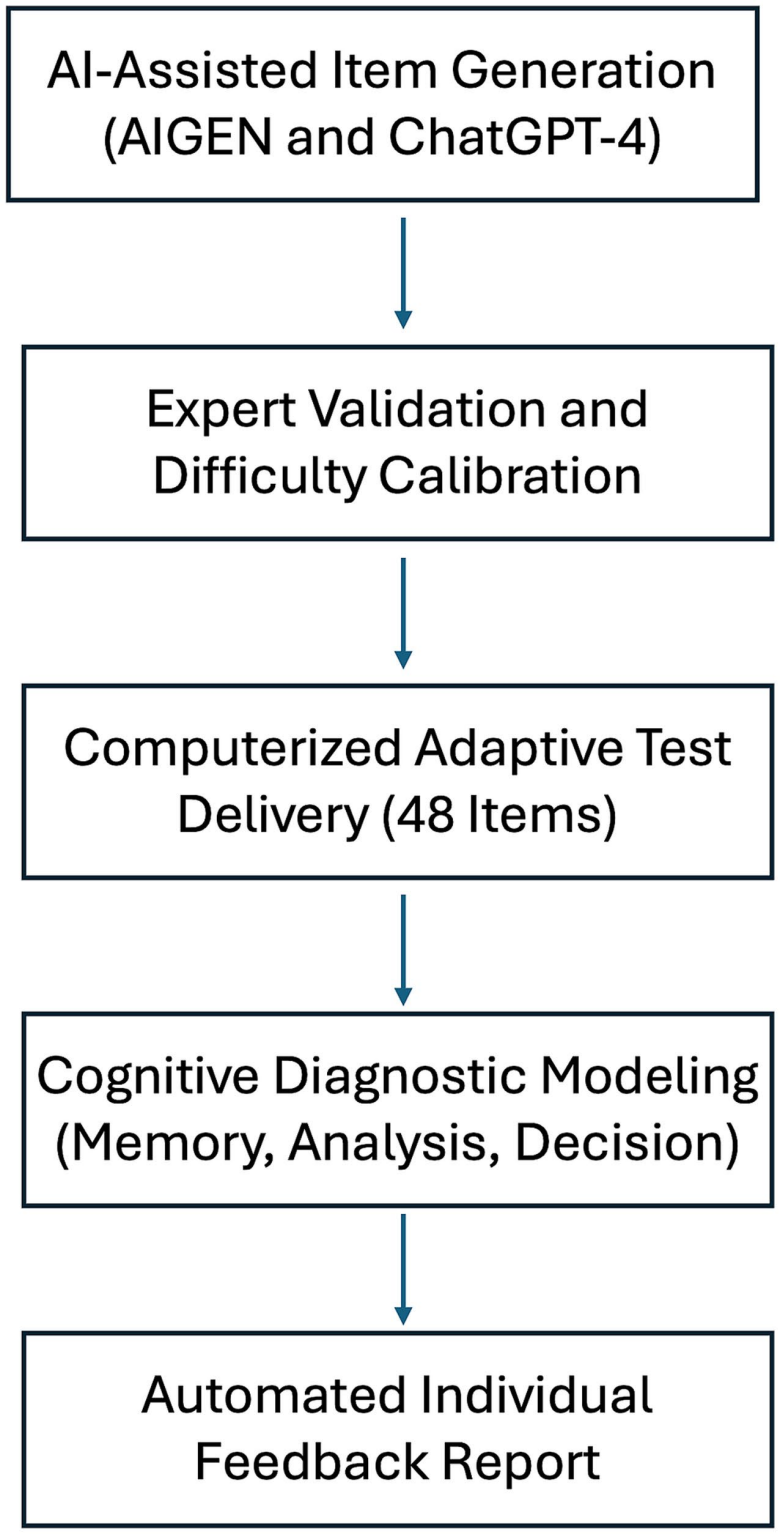
Workflow of AI-assisted item generation, validation, and calibration. Items were first generated by ChatGPT-4 guided by cognitive models and blueprint alignment, categorized by cognitive domain (memory, analysis, decision) using dual independent coding (*κ* = 0.76), validated by expert review, and integrated with pre-calibrated OAIPT items. Final items were assigned initial difficulty indices (−3 to +3 IRT scale) and empirically validated during administration.

An item bank was developed using Automated Item Generation (AIGen) techniques, guided by cognitive models aligned with the exam blueprint (Leighton and Gierl, 2007; Falcão et al., 2023; Gierl et al., 2022; Falcão et al., 2022). The overall workflow of AI-assisted item generation, expert validation, and calibration is illustrated in Figure 1. Each item was also categorized according to three cognitive diagnostic models based on the NBME framework (Billings et al., n.d.): memory (questions that require recall of factual information), analysis (questions that demand interpretation of clinical information), or decision (questions that require synthesis and clinical critical decision). To ensure consistency, two independent raters with experience in medical education and assessment independently classified all items according to these categories. Inter-rater reliability for initial classification was substantial (Cohen’s *κ* = 0.76), indicating good agreement. Discrepancies were resolved through discussion until consensus was achieved.

Additionally, a retrospective double-blind classification of a random sample of 25 items by two external raters who were not involved in the original coding. Inter-rater reliability for this retrospective validation was Cohen’s *κ* = 0.68, indicating substantial agreement and supporting the consistency of domain assignment. In addition to newly generated items, calibrated items from the existing bank of the Online Adaptive International Progress Test (OAIPT) were incorporated, taking advantage of their known difficulty parameters and item quality. For the newly created items, difficulty indices (ranging from −3 to +3 on the IRT scale) were initially estimated using ChatGPT-4 (OpenAI, web interface) with the following structured prompt: “Given the previous examples with their respective difficulty indices, estimate the expected difficulty of the following multiple-choice question for 5th-year medical students in a summative exam.” Each AI-generated estimate and rationale were reviewed by two content experts for accuracy and consistency. Items were accepted only when both experts agreed that (a) the content was clinically correct, (b) the reasoning aligned with the intended cognitive domain, and (c) the predicted difficulty was plausible compared with similar calibrated items. Disagreements were resolved through discussion. This process produced an initial difficulty calibration for new items before empirical validation during test administration. To enhance transparency, representative examples of each cognitive domain are provided in Supplementary material 1, allowing readers to evaluate the appropriateness of domain assignment.

### Platform setup

The digital assessment platform QuizOne^®^ was used to deliver CAT via its dedicated module (Rice et al., 2022). This platform enabled dynamic test adaptation based on individual student responses. Weighted Likelihood (WL) method was used as the theta estimator, providing robust estimates of student ability even with a small number of items. For the selection of subsequent items, the platform applied the Maximum Fisher Information (MFI) criterion, which chooses the next item that maximizes the expected information gain at the student’s ability level (Chang and Ying, 1996).

### Formative CAT exam design

A formative Computerized Adaptive Test (CAT) consisting of 48 multiple-choice questions (MCQs) was created for this study and made available to students 3–5 days before the summative Progress Test of the Surgical Curricular Unit. The CAT was delivered through the platform and integrated Cognitive Diagnostic Modeling (CDM) to classify each item by cognitive domain—memory, analysis, or decision. The platform was programmed to present one item from each cognitive domain across 16 surgical topics defined in the exam blueprint: Urology, Trauma, Abdominal Wall, Orthopedics, Otorhinolaryngology, Ophthalmology, Neurosurgery, Hepatobiliopancreatic, Esophagogastric, Endocrine and Breast, Colorectal, and Vascular Surgery. Each student therefore, completed exactly 48 items, as the adaptive algorithm was configured with a fixed-length stopping rule to ensure comparable duration and psychometric precision across participants. The formative CAT aimed to reinforce learning by identifying specific cognitive gaps and providing individualized feedback on students’ performance profiles. While there was curricular overlap in learning objectives, the specific items used in the formative CAT were distinct from those included in the summative exam, preventing direct content duplication.

Additionally, the authors collected the reports of the previous two exams of the surgical curricular unit (Exam 1 and Exam 2) done by the same student sample.

### Participants

All the students who enrolled in the surgical curricular unit were invited to participate in the formative exam. A convenience sample of 116 volunteer students completed the formative CAT exam.

### Data collection and analysis

Item-level performance data and overall test scores were extracted from the platform. Statistical analysis was performed using JASP (version 0.18.1).

Before inferential testing, the distribution of all continuous variables was assessed using the Shapiro–Wilk test to evaluate assumptions of normality. Normality was tested with the Shapiro–Wilk test. When assumptions of normality were violated, non-parametric tests were applied (Spearman’s *ρ* for correlations, Mann–Whitney *U* for between-group comparisons, Kruskal–Wallis *H* for multiple groups). Linear regression assumptions were examined by inspecting residual plots and variance inflation factors. All analyses were independently verified by two authors to ensure consistency and accuracy of results. To examine relationships between cognitive performance indicators and summative outcomes, we conducted correlation analyses between final CAT scores, CDM sub-scores, and summative exam grades. Group comparisons were performed to assess differences between students who completed the formative CAT exam and those who did not. Additionally, ANCOVA models were used to evaluate the independent effect of CAT participation on summative performance, adjusting for prior academic performance. Finally, simple linear regressions were performed to identify predictors of summative exam scores, including the contribution of specific CDM sub-scores and overall test performance. Statistical significance was set at *p* < 0.05.

### Feedback delivery

Upon completion of the formative CAT, each student received an individualized automated feedback report generated by the platform. The report began with a brief explanation of the standardized scoring system (mean = 500, SD = 100) and an illustrative graph clarifying how to interpret scores (e.g., 600 = 1 SD above average). Subsequent pages presented tables and bar charts displaying the student’s standardized scores across multiple dimensions: 1) System categories (e.g., respiratory, digestive, musculoskeletal); 2) Surgical disciplines (e.g., hepatobiliopancreatic, colorectal, trauma, vascular); 3) Medical competencies (diagnosis, treatment, scientific principles, management, pathophysiology); 4) Cognitive domains (memory, analysis, decision).

### Satisfaction questionnaire

At the end of the curricular unit, participating students completed a satisfaction questionnaire assessing the usability, perceived usefulness, and impact of CAT and CDM on their learning process. The questionnaire consisted of nine Likert-scale items (1 = least positive; 5 = most positive) and two open-ended questions addressing the most positive and least positive aspects of the test and feedback. However, the response rate was insufficient (13%, 15 of 116 participants) to allow meaningful analysis; therefore, these data were not included in the present report.

### Ethical commission approval

This study was conducted following the ethical principles outlined in the Declaration of Helsinki. Ethical approval was obtained from the Ethics Committee for Research in Life and Health Sciences, University of Minho. Participation was voluntary, and all students provided informed consent before inclusion in the study. No identifiable personal data was collected. Participants were informed that their academic evaluation would not be influenced by their decision to participate or by their performance in the formative assessments. Data were stored securely and used exclusively for research purposes.

## Results

The final item bank used in the CAT exam was composed of 150 MCQ items, 84 extracted from the previously calibrated database (OAIPT) and 66 created by faculty members, experienced in item writing. From that item bank, the software developed an individual customized exam with 48 questions, tailored to the student’s level of competence, with a matching blueprint of the same topics as the progress test and the previous surgical curricular unit. The 48 questions were equally divided between the three different diagnostic models: decision, memory, and analysis.

**Figure 2:**
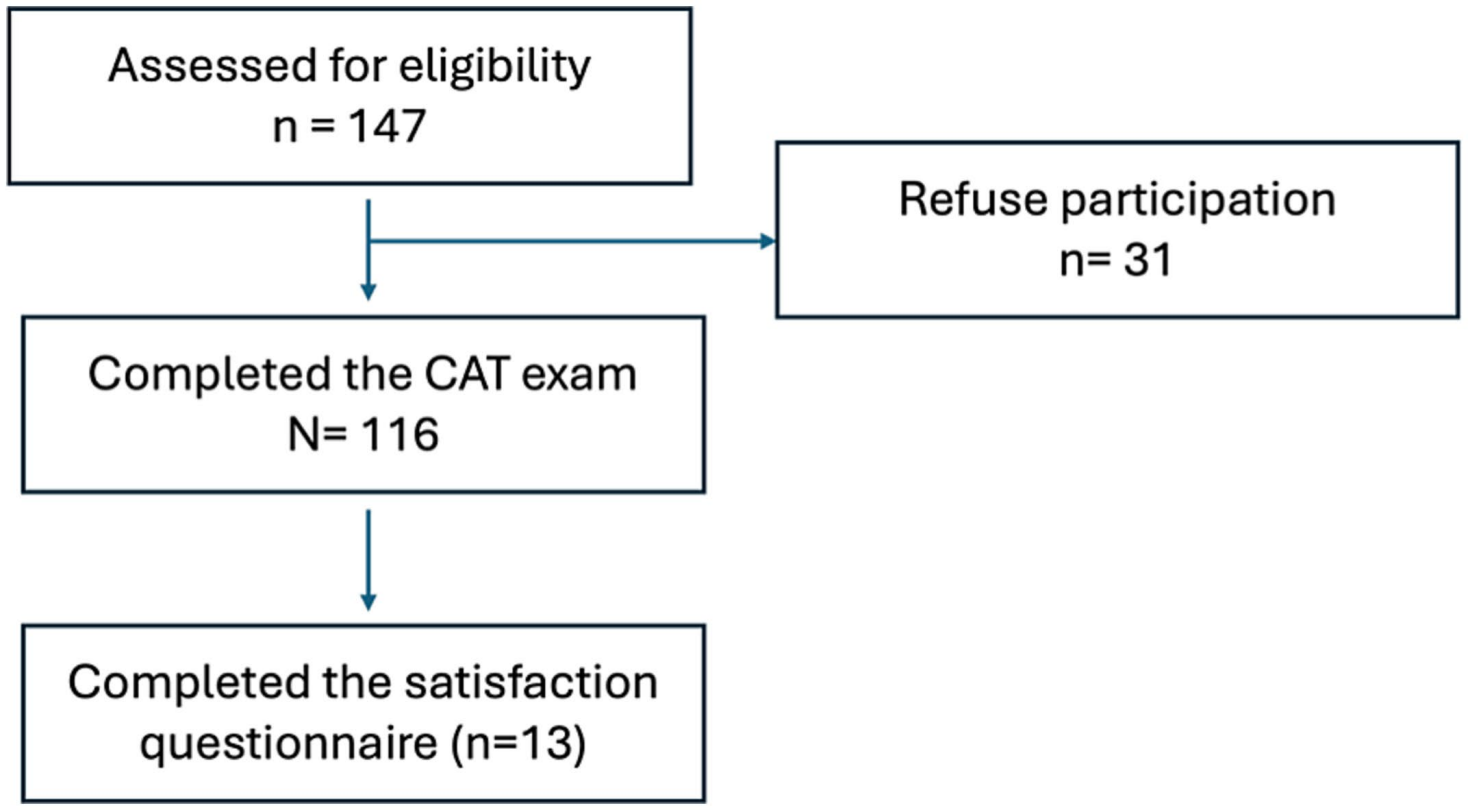
CONSORT-style flowchart illustrating participant inclusion and attrition. Of the 147 students initially enrolled in the Surgical Curricular Unit, 116 voluntarily completed the formative Computerized Adaptive Test (CAT). Thirty-one students did not participate. Among the CAT participants, 15 completed the post-course satisfaction survey.

A total of 147 students were included in the analysis, of whom 116 completed the formative CAT. The participant inclusion process and attrition are illustrated in Figure 2. A post-hoc power analysis for the ANCOVA comparing CAT participants (*n* = 116) and non-participants (*n* = 31), with *α* = 0.05, *f* = 0.173 (derived from *η*^2^ = 0.029) and two covariates, indicated approximately 0.49 power to detect this effect size. This suggests limited sensitivity for small effects, consistent with the exploratory nature of the study.

Normality was evaluated using the Shapiro–Wilk test. Most variables, including Progress Test, Exam 1, and Exam 2, and CDM-specific measures, deviated from normality in at least one group (*p* < 0.05). Therefore, non-parametric tests were applied when assumptions were not met. As expected, grades from the CAT exam followed a normal distribution. Table 1 summarizes the descriptive statistics for all key variables, including the overall CAT score, cognitive sub-scores, and summative exam results.

**Table 1:** Descriptive statistics for key variables, including mean, standard deviation, and observed range values for the formative CAT total score, cognitive domain sub-scores, and summative assessments.

Variable	Mean	SD	Minimum	Maximum
Final CAT score	589.95	46.44	447.00	698.00
Skill–Memory	604.79	54.87	446.00	713.00
Skill–Analysis	564.94	57.71	434.00	694.00
Skill–Decision	551.11	33.05	436.00	671.00
Progress Test score	14.91	2.16	8.80	18.20
Exam 1 score	12.98	2.07	5.70	17.10
Exam 2 score	12.49	2.47	6.20	17.20

**Figure 3:**
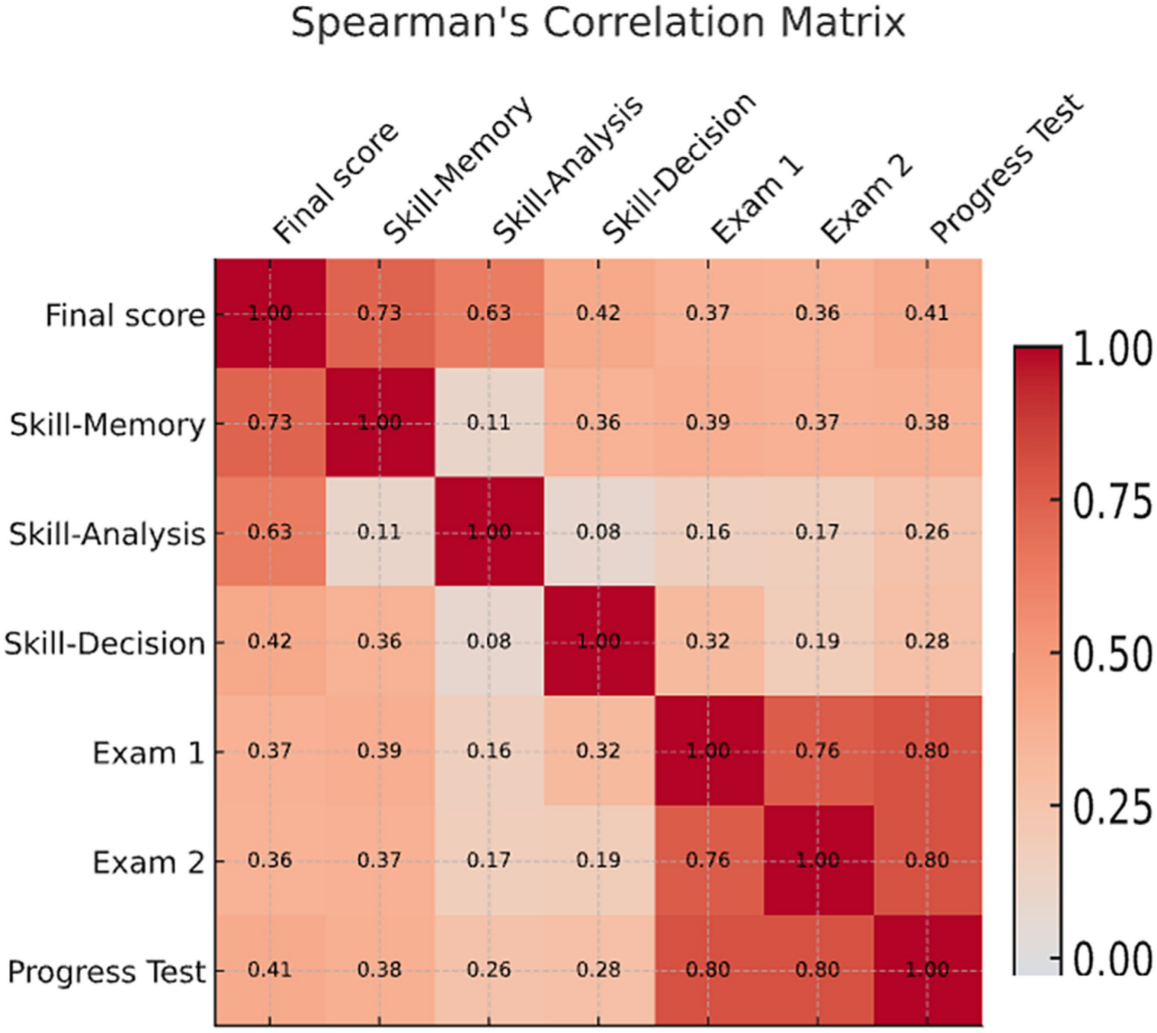
Spearman’s correlation matrix between adaptive test performance, cognitive diagnostic dimensions, and academic outcomes. The heatmap shows correlations between the Computerized Adaptive Test (CAT) total score, Cognitive Diagnostic sub-scores (Skill–Memory, Skill–Analysis, Skill–Decision), and summative exam results (Exam 1, Exam 2, and Progress Test). Darker red shades indicate stronger positive correlations, while blue indicates negative associations. All displayed coefficients are significant at *p* < 0.05.

Spearman’s rank correlations were computed to examine the relationships between the final CAT score, cognitive diagnostic sub-scores (Skill–Memory, Skill–Analysis, Skill–Decision), and academic outcomes (Exam 1, Exam 2, and Progress Test). Results are detailed in Table 2 and visually represented in Figure 3.

**Table 2:** Multiple Linear regression analyses evaluating the predictive value of the final CAT score and cognitive diagnostic sub-scores (Skill–Memory, Skill–Analysis, Skill–Decision) on Progress Test performance.

Predictor	*β* (standardized)	95%CI	*t*	*p*
Skill–Memory	0.41	[0.002, 0.029]	2.22	0.29
Skill–Analysis	0.25	[0.003, 0.021]	1.48	0.143
Skill–Decision	0.05	[−0.008, 0.015]	0.58	0.565
Final CAT score	−0.05	[−0.0025, 0.020]	−0.20	0.845

Model: *R*^2^ = 0.23, adjusted *R*^2^ = 0.20, *F*(4,107) = 7.98, *p* < 0.001.

The final CAT score showed strong correlations with Skill–Memory (*ρ* = 0.73, *p* < 0.001) and Skill–Analysis (*ρ* = 0.63, *p* < 0.001), and a moderate correlation with Skill–Decision (*ρ* = 0.42, *p* < 0.001).

Regarding academic outcomes, the Progress Test was moderately correlated with Skill–Memory (ρ = 0.38, *p* < 0.001) and showed weaker associations with Skill–Analysis (*ρ* = 0.26, *p* = 0.006) and Skill–Decision (*ρ* = 0.28, *p* = 0.003). Both Exam 1 and Exam 2 correlated more strongly with Skill–Memory than with the other sub-scores. Additionally, Exam 1, Exam 2, and the Progress Test were highly intercorrelated (*ρ* ≈ 0.8, *p* < 0.001), indicating consistent performance across summative assessments.

**Figure 4:**
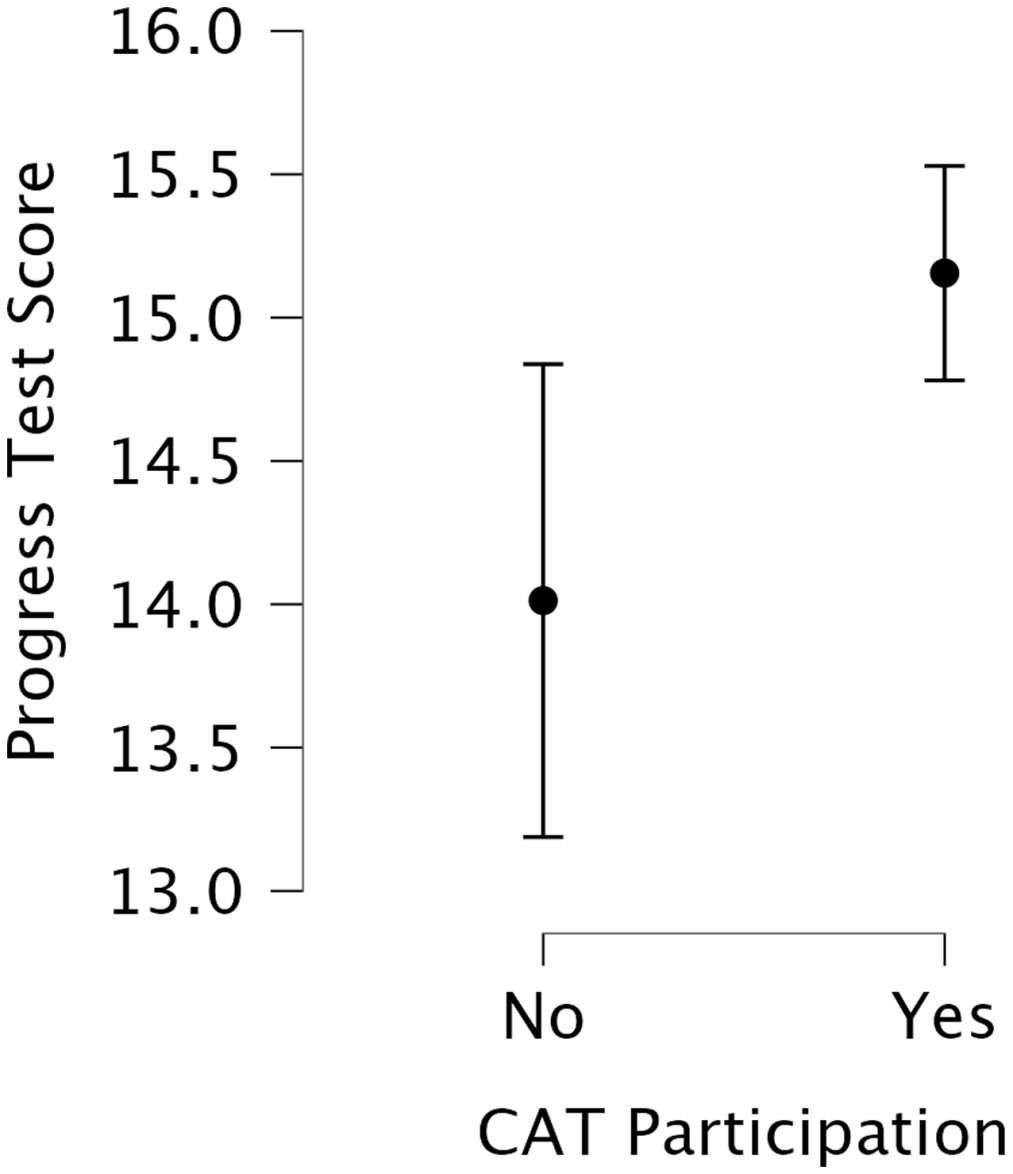
Box plot comparing Progress Test scores between students who participated in the formative Computerized Adaptive Test (CAT) and those who did not. CAT participants achieved significantly higher scores on the summative Progress Test (*U* = 1,311, *p* = 0.021, rank-biserial correlation = −0.27). Error bars represent standard deviations.

A Mann–Whitney test revealed that CAT participants scored significantly higher on the Progress Test than non-participants (*U* = 1,311, *p* = 0.021, rank-biserial correlation = −0.271). The differences in Exam 1 (*U* = 1392.5, *p* = 0.054) and Exam 2 did not differ significantly between groups (*U* = 1,466, *p* = 0.115). Figure 4 illustrates the group comparison of Progress Test scores between students who completed the formative CAT and those who did not.

An ANCOVA was conducted to evaluate the impact of participation in the formative CAT exam on the summative score. After adjusting for prior performance (Exam 1 and Exam 2), participation in the formative CAT exam had a statistically significant effect on the Progress Test, *F*(1, 143) = 4.239, *p* = 0.041, *η*^2^ = 0.029. Both Exam 1 (*F* = 35.835, *p* < 0.001) and Exam 2 (*F* = 48.388, *p* < 0.001) were also significant predictors of performance, indicating that earlier academic performance was strongly associated with the outcome. These findings suggest that the formative exam were associated with improved performance beyond what could be explained by prior academic achievement alone. Figure 4 illustrates the regression relationships between CAT performance metrics and summative Progress Test results.

A series of linear regressions were conducted to evaluate the predictive value of the final CAT score and each cognitive diagnostic sub-score (Skill–Memory, Skill–Analysis, and Skill–Decision) on Progress Test performance.

The model using the final CAT score as a predictor was statistically significant, *F*(1, 114) = 25.364, *p* < 0.001, explaining 18.2% of the variance in Progress Test scores (*R*^2^ = 0.182). The final CAT score emerged as a significant positive predictor (*β* = 0.427, *p* < 0.001), indicating that higher adaptive test performance was associated with improved summative outcomes.

**Figure 5:**
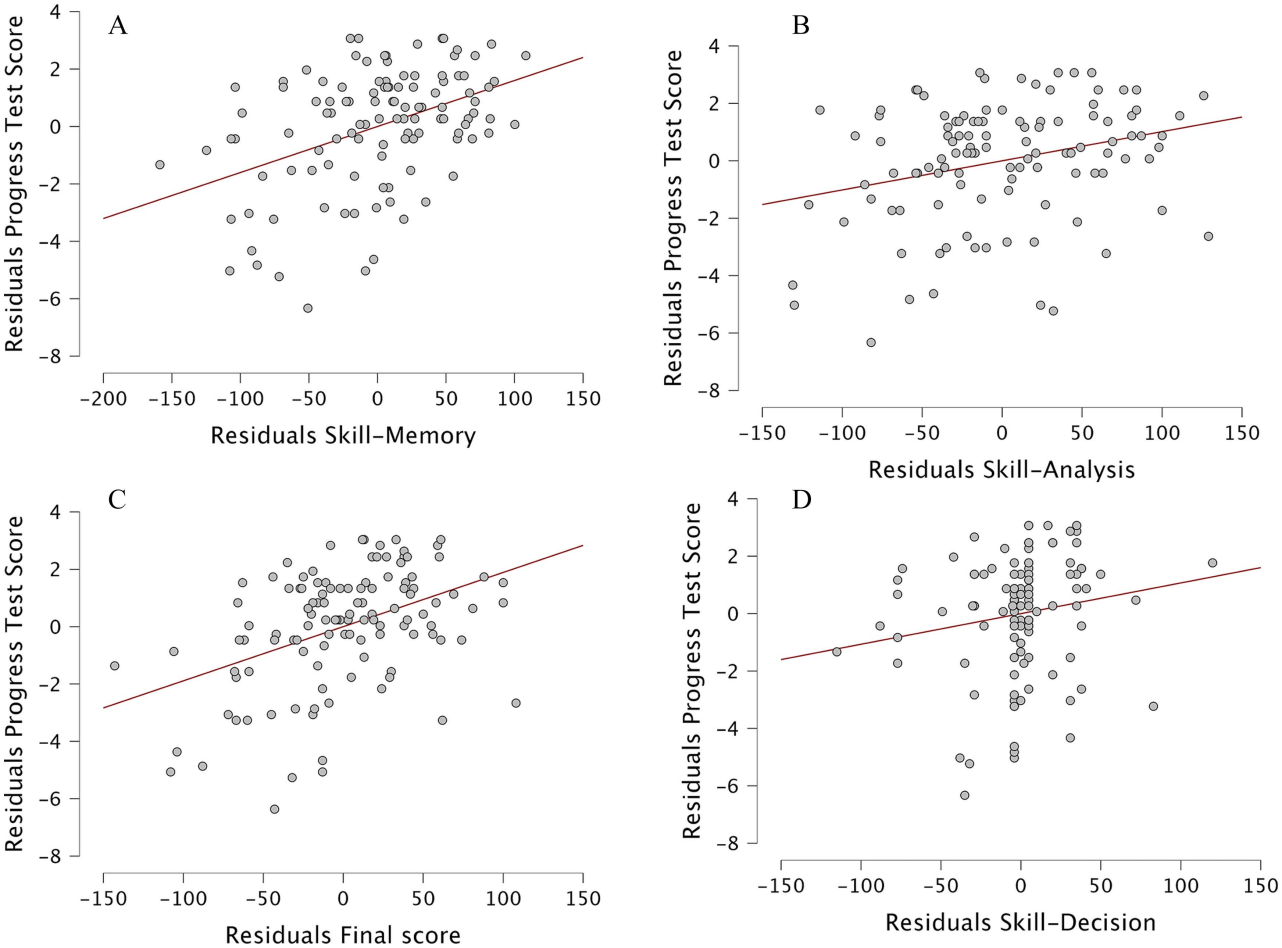
Scatterplots showing relationships between formative Computerized Adaptive Test (CAT) performance and summative Progress Test outcomes. Each panel displays standardized residuals from linear regression models with fitted regression lines and 95% confidence intervals. **(A)** Skill–Memory, **(B)** Skill–Analysis, **(C)** Final CAT score, and **(D)** Skill–Decision. Among the predictors, Skill–Memory showed the strongest positive association with Progress Test performance, while the other domains exhibited weaker or non-significant trends.

When each cognitive skill domain was analyzed separately, Skill–Memory was the strongest predictor of Progress Test performance, *F*(1, 110) = 24.196, *p* < 0.001, accounting for 18.0% of the variance (*R*^2^ = 0.180; *β* = 0.425, *p* < 0.001), indicating a moderate positive association between memory skills and Progress Test performance. Skill–Analysis also significantly predicted Progress Test scores, *F*(1, 110) = 9.574, *p* = 0.003, though with a smaller effect size (*R*^2^ = 0.080; *β* = 0.283, *p* = 0.003). In contrast, Skill–Decision explained only 2.9% of the variance and was not a significant predictor (*R*^2^ = 0.029; *β* = 0.170, *p* = 0.072). However, when all domains were entered simultaneously into a multiple regression model, only *Skill–Memory* remained a significant independent predictor (*β* = 0.41, *p* = 0.029), confirming its dominant role after controlling for intercorrelations among cognitive domains (Table 1). Figure 5 illustrates the regression relationships between CAT performance metrics and summative outcomes.

## Discussion

This study explored the use of CAT combined with CDM as a formative assessment tool in undergraduate surgical education. A distinctive feature of the intervention was the integration of AI into both item development and feedback generation, allowing efficient test calibration, individualized difficulty estimation, and automated reporting with minimal faculty workload. These findings align with the growing evidence that AI-driven assessment systems can enhance precision, scalability, and personalization in medical education (Pohn et al., 2025; Shaw et al., 2025; Gordon et al., 2024; Mir et al., 2023; Sunmboye et al., 2025; Gomez et al., 2025).

The association between participation in the formative CAT–CDM exam and higher performance in the subsequent summative Progress Test suggests that adaptive, feedback-oriented assessments may promote more effective study strategies. The multiple regression analysis, including all cognitive domains and the total CAT score, confirmed that only *Skill–Memory* remained a significant independent predictor of summative performance, whereas *Skill–Analysis* and *Skill–Decision* did not contribute additional explanatory value once intercorrelations were controlled for. This finding indicates that the predictive relationship between analytical and decision-making domains and summative performance is largely shared with memory-based competence. It also reinforces the interpretation that current multiple-choice examination formats primarily reward factual recall rather than complex reasoning or integrative decision-making. These results highlight the need for assessment strategies capable of isolating higher-order cognitive processes from underlying knowledge recall. One possible explanation is that items categorized as decision may not have captured the full complexity of real-world clinical reasoning. As highlighted in previous work, problem-solving in surgery often depends on context, uncertainty, and prioritization rather than discrete knowledge application (Ross et al., 2022; Crebbin et al., 2013) Multiple-choice questions, even when well constructed, are limited in their ability to elicit such integrative reasoning. In contrast, memory items, more closely aligned with the structure of summative exams, may show stronger statistical relationships simply because both assessments rely on similar cognitive processes. This alignment may reflect systemic bias toward factual recall in traditional testing, emphasizing the need for new formats that authentically measure complex reasoning, such as rich clinical vignettes, branching scenarios, or virtual patients (Gomez et al., 2025; Billings et al., n.d.; Rice et al., 2022) Beyond item design limitations, several alternative explanations merit consideration. First, decision-making competence may not yet be sufficiently developed in fifth-year medical students to exhibit measurable variance, given that authentic clinical decision-making typically matures during postgraduate training. Second, the near-zero correlation between analysis and decision domains (*ρ* = 0.08) suggests potential overlap or misalignment within the cognitive classification framework itself, indicating that the current three-domain model may have limited discriminant validity. Third, the summative Progress Test used as the external criterion primarily measures factual and analytical reasoning, which may not adequately fully capture the situational judgment or uncertainty management, considered core features of surgical decision-making. Forth, decision-making items may demand context integration and abstraction beyond what can be effectively assessed in a brief, text-based MCQ, causing students to rely on pattern recognition rather than deliberate reasoning. Fifth, the weak association might also reflect a misalignment between formative and summative constructs: whereas the CAT–CDM aimed to assess applied reasoning, the Progress Test could be predominantly capturing factual recall, thereby reducing shared variance by design. These combined factors likely explain the absence of significant associations and underscore that current multiple-choice formats are inherently constrained in representing complex cognitive processes such as risk–benefit reasoning, ethical trade-offs, and context-specific prioritization. As such, the null finding highlights an important boundary in the construct validity of decision-making assessment and identifies an area for future instrument development and validation.

From an educational perspective, these results have practical implications for curriculum design. Although statistically reliable, the effect sizes were modest, suggesting that the practical impact of short formative interventions may be incremental rather than transformative. If recall remains the main predictor of summative success, surgical educators risk over-emphasizing rote knowledge at the expense of reasoning and decision-making competence. Integrating adaptive cognitive diagnostics into teaching could help identify students who rely predominantly on memorization and guide them toward deliberate practice in interpretation and judgment. Educators should consider coupling CAT-CDM data with simulation or case-based discussions, transforming feedback into structured remediation plans. In parallel, AI-supported feedback dashboards could provide students with a dynamic map of their evolving cognitive profile, encouraging self-regulated learning and early correction of deficiencies. By integrating these tools, surgical curricula could progressively shift from knowledge reproduction to diagnostic reasoning and decision-making mastery.

Beyond its psychometric contribution, the present study provides several educational insights. The CAT–CDM framework aligns closely with principles of adaptive learning, in which instructional content and assessment dynamically adjust to each learner’s ability level, which we believe to be key to learning. By identifying specific cognitive domains requiring reinforcement, adaptive testing provides personalized diagnostic feedback that can guide self-regulated learning. Students can use the domain-specific results to direct their study strategies toward weaker areas, engage in deliberate practice, and monitor progress over time. From an instructional standpoint, such diagnostic information enables educators to allocate remediation resources more efficiently and to tailor teaching toward common cognitive gaps.

Furthermore, the integration of AI-assisted item generation and feedback represents a scalable model for formative assessment with minimal faculty workload, consistent with the growing emphasis on feedback as a continuous, learner-driven process rather than an episodic event. By converting performance data into interpretable cognitive profiles, this approach helps close the feedback loop and fosters reflection, autonomy, and iterative improvement.

Several limitations must be acknowledged. First, participation in the formative exam was voluntary, introducing potential selection bias, as more motivated students may have been more likely to participate. Although all students from the same curricular unit were invited to participate, and overall participation was high (79%), voluntary participation may have led to self-selection bias, with more motivated or academically stronger students being overrepresented among CAT participants. This possibility should be considered when interpreting the association between formative and summative performance since it restricts the ability to establish causal inferences; therefore, the observed differences should be interpreted as associations rather than direct effects of the intervention. This short interval of 3–5 days before the summative Progress Test mirrors real-world preparation patterns in which students consolidate learning shortly before assessment. The aim was not to measure long-term knowledge retention but to determine whether adaptive feedback could inform final revision strategies. Future studies will extend the interval between formative and summative assessments to investigate sustained learning effects and behavioral change over time. Second, this was a single-center study, potentially limiting applicability to other educational contexts. Finally, the study focused on short-term outcomes; future research should examine whether the observed benefits persist in long-term knowledge retention and clinical performance. Although initial inter-rater reliability for cognitive classification was substantial (*κ* = 0.76), and retrospective double-blind validation of a sample of items demonstrated similarly substantial agreement (*κ* = 0.68), some residual subjectivity in distinguishing analysis from decision items cannot be fully excluded. This issue is particularly relevant given the weaker associations observed in the Skill–Decision domain. Future research should include independent expert classification of the entire item bank to further strengthen the construct validity of cognitive domain distinctions.

Future research should employ randomized or crossover designs to confirm efficacy, explore the durability of learning effects, and examine the longitudinal impact of adaptive feedback on self-regulated learning. Establishing causal links between adaptive testing, feedback quality, and performance improvement would provide stronger evidence for scaling this approach. Integrating the CAT–CDM model with simulation-based or virtual-patient environments could enhance authenticity and allow assessment of higher-order decision-making under uncertainty—an essential but often under-evaluated component of surgical competence. Expanding item banks with scenario-driven and branching questions may further strengthen construct validity for complex reasoning. Ultimately, such investigations could contribute to a more holistic and adaptive assessment ecosystem encompassing the cognitive, technical, and non-technical dimensions of surgical education.

## Conclusion

This study found that the use of Computerized Adaptive Testing (CAT) combined with Cognitive Diagnostic Modeling (CDM), supported by AI-based item generation and automated feedback, was associated with higher performance in a subsequent summative assessment. Although decision-making skills were underrepresented in predictive models, the results highlight the need for curricular strategies that better promote higher-order cognitive processing in surgical education. Within the acknowledged methodological limitations, AI-enhanced CAT–CDM emerges as a promising approach for delivering meaningful cognitive feedback and fostering data-informed learning in medical training.

## Data Availability

The raw data supporting the conclusions of this article will be made available by the authors, without undue reservation.
