# Supplementary material for: AI-enhanced adaptive testing with cognitive diagnostic feedback and its association with performance in undergraduate surgical education: a pilot study
Source: Front Behav Neurosci. 2026 Jan 6;19:1735237. doi: 10.3389/fnbeh.2025.1735237 (PMC12816294; doi:10.3389/fnbeh.2025.1735237)
Supplement: Supplementary file 1 [file Table_1.DOCX]

Question 1) Analysis:

A 54-year-old man presents to the clinic with a painless mass on the anterior abdominal wall that he first noticed about 3 months ago. The mass increases in size with straining, such as coughing or lifting heavy objects, and decreases when lying down. He reports no significant pain or discomfort, nausea, vomiting, or changes in bowel habits. The patient has a history of obesity and hypertension but no prior surgeries. Physical examination reveals a soft, reducible protrusion located in the midline above the umbilicus. The overlying skin shows no signs of inflammation.

Which of the following is the most likely diagnosis?

Rectus abdominis diastasis

Epigastric hernia

Umbilical hernia

Subcutaneous lipoma

Intra-abdominal tumor

Question 2) Memory

A 34-year-old man presents to the emergency department with sudden eye pain and vision loss in the right eye after a workplace accident. He was using a cutting tool when a metal fragment struck his eye. On examination, there is marked tearing and photophobia in the right eye, with vision reduced to light perception. Inspection reveals a corneal laceration with iris prolapse through the defect. Intraocular pressure is decreased in the affected eye.

Which of the following structures forms the outermost layer of the eyeball wall and provides structural protection?

Cornea

Choroid

Sclera

Vitreous humor

Retina

Question 3) Decision

A 4-year-old girl is brought to the clinic with 3 days of fever, rhinorrhea, and nasal congestion. She completed a course of oral antibiotics for an ear infection 3 weeks ago and was noted to have a middle ear effusion during a routine visit last week. There is no history of other infections or medical conditions. She has no allergies. Both parents smoke cigarettes, and her two older siblings had tympanostomy tubes placed as infants.

Her temperature is 39.4 °C. The patient is irritable but easily consoled by her mother. Examination of the external ears is normal, and the external auditory canals are patent. Otoscopy shows bilateral bulging, erythematous tympanic membranes with reduced mobility on insufflation. The oropharynx is normal without lesions, and lung auscultation is clear.

Which of the following is the most appropriate next step in management of this patient?

Oral antibiotics

Supportive care and observation

Nasopharyngeal viral PCR testing

Tympanocentesis and culture

Temporal bone computed tomography (CT)
