# Supplementary material for: AI-enhanced adaptive testing with cognitive diagnostic feedback and its association with performance in undergraduate surgical education: a pilot study
Source: Front Behav Neurosci. 2026 Jan 6;19:1735237. doi: 10.3389/fnbeh.2025.1735237 (PMC12816294; doi:10.3389/fnbeh.2025.1735237)
Supplement: Supplementary file 2 [file Table_2.DOCX]

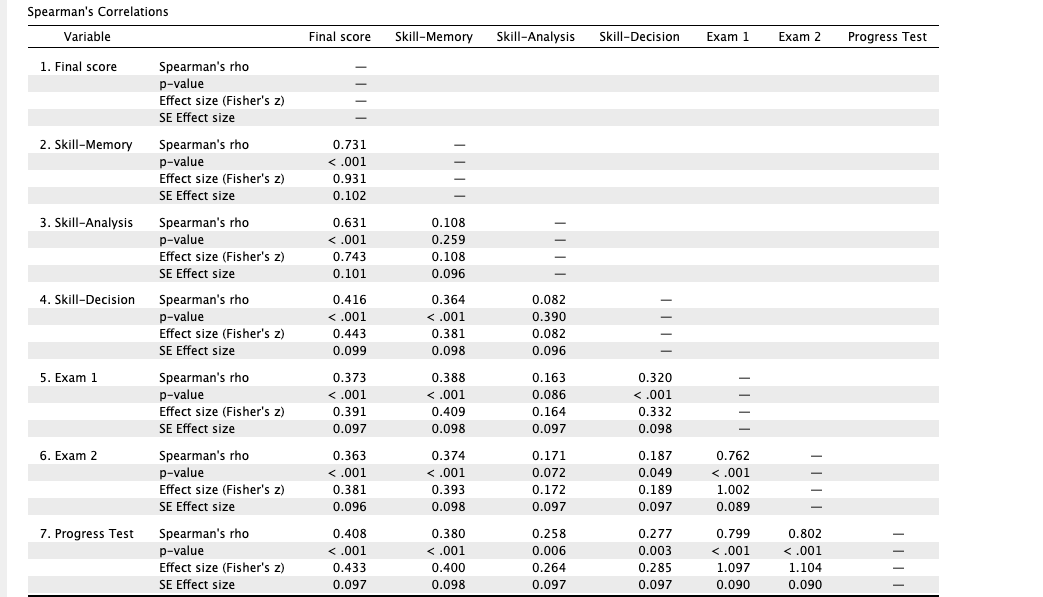


Supplementary Table 1 - Correlation matrix between Computerized Adaptive Test (CAT) total score, Cognitive Diagnostic sub-scores (Memory, Analysis, Decision), and academic outcomes (Exam 1, Exam 2, and Progress Test).
